# Pure Milk or Raw Milk? A Sidelight on the Milk Problem

**Published:** 1914-02-21

**Authors:** 


### PURE MILK OR RAW MILK?

### A Sidelight on the Milk Problem.

The pure-milk problem is one of the most pressing which confronts the various agencies—voluntary, municipal, or governmental—which seek to minimise the excessive infantile mortality of urban communities at the present day. Thirty years ago a variety of artificial foods rose rapidly, mainly through lavish advertisement, into a popularity as milk substitutes which has not yet by any means been destroyed, notwithstanding the preaching of the medical profession. Then came the reign of sterilised milk, as a preferable alternative for young infants to starchy rickets-producing patent foods or to milk infected with the germs of diarrhœa or tuberculosis. Presently it was apparent that sterilised milk has disadvantages too.

When sterilised milk fell into disfavour, pasteurised milk had a vogue: milk, that is to say, which has been heated to a temperature far short of the boiling point, but sufficient to destroy those kinds of microbes which are most frequently the sources of infantile mortality. By this process it was expected and hoped that coagulation of milk albumins and the destruction of ferments, anti-bodies, and similar useful components, would be avoided. But in course of time experience proved that even pasteurised milk is not satisfactory as a substitute food for infants, and that it falls far short of milk which is *pure* and *fresh*. To procure milk which can be relied on for these two qualities is exceedingly difficult in this country, though it is not now nearly so difficult as it was a few years back. But it is still, when procurable, relatively expensive; and this puts it more or less out of reach of the bulk of the population. Until the improvement of the present, in general, low standards of purity of the milk consumed by the public, whether infant or adult, is brought about, there are many people, including medical men of standing and reputation, who advise parents to buy either pasteurised milk or some of the newer milk substitutes, which are far more scientifically devised and carefully prepared than their forerunners of a generation ago.

### The Weak Point in Pasteurisation.

The failure, or comparative failure, of pasteurised milk to realise the expectation formed of it, is probably explicable by some recent researches published in *American Medicine* by Dr. Ralph Vincent. This observer points out that there is no accepted standard of temperature, or of length of exposure to it, by which "pasteurised" milk can be judged. Originally pasteurisation meant merely heating to boiling point, not under pressure, on each of three successive days. Then a temperature of 180 deg. Fahr. was held to be the right one, though even then there was no agreement as to how long it should be maintained. Then Freeman designed his pasteuriser to heat milk up to 167 deg. Fahr., and later modified it to 155 deg. as he found 167 deg. too high. The most recent development is to "pasteurise" for twenty minutes at 140 deg. Fahr.

Against milk thus treated Dr. Vincent lays two objections. One is that any method of pasteurisation which effectually and certainly destroys the "pathogenic" organisms (that is, the germs of those diseases which impure milk may convey to those who drink it) also destroys the organisms typical of pure milk. The latter organisms, according to this new view, do much to hinder the growth of the pathogenic kinds, and are, therefore, of positive value in milk. The second objection is that the bacterial growth which takes place in pasteurised milk, which is afterwards exposed to the air and incubated at body temperature, is wholly different from that which occurs in raw milk in the same circumstances. In other words, pasteurisation, though it may destroy harmful microbes for the time being, makes the milk much more likely to "go wrong."

In support of these propositions, this author quotes his own researches as embodied in recent books from his pen. As these are of much interest, we may condense them as follows. Pure raw milk incubated at blood heat for eight hours shows a luxuriant growth of a germ known as *Streptococcus lacticus*. This microbe does no harm to infants, and as a matter of fact it is even present in the milk which an infant takes direct from its mother's breast. Not only so, its development almost prevents the growth of other microbes. If milk is heated to 170 deg. Fahr. for ten minutes, and then incubated, in forty-eight hours it is stinking, and swarming with a dangerous germ known as *Bacillus putrificus*. Milk heated to 190 deg. Fahr. for three minutes and incubated for forty-eight hours is found to be curdled and to contain large numbers of a microbe which is responsible for severe and often fatal infantile diarrhœa; and worse still happens in milk which has been actually boiled (212 deg. Fahr.) for three minutes, for after incubation it contains several kinds of deadly micro-organisms.

None of these disease-producing germs will grow, fortunately, in the stomach or intestines of a child which is being fed on raw milk, for the beneficent *Streptococcus lacticus* prevents them from doing so. But when an infant is systematically fed on pasteurised milk, the danger of their development is always present; because the lactic cocci are delicate and soon die out. Thus Dr. Vincent concludes that fresh supplies of these protective germs are one of the constant special requirements of a baby. When they fail to flourish, acute or chronic disease gets a chance to arise. The actual outbreak is frequently preceded by a colon toxæmia, which he holds is a necessary intermediate stage between the normal and the abnormal states.

The practical import of these researches, if their accuracy and the validity of the conclusions founded on them is admitted, is the reassertion of the paramount claims of raw cow's milk as the one dependable substitute for mother's milk.

